# An Opportunity for Cognitive Task Analysis in Neonatal Resuscitation

**DOI:** 10.3389/fped.2019.00356

**Published:** 2019-08-27

**Authors:** Emily C. Zehnder, Brenda H. Y. Law, Georg M. Schmölzer

**Affiliations:** ^1^Neonatal Research Unit, Centre for the Studies of Asphyxia and Resuscitation, Royal Alexandra Hospital, Edmonton, AB, Canada; ^2^Department of Pediatrics, University of Alberta, Edmonton, AB, Canada

**Keywords:** neonatal resuscitation, delivery room, cognitive task analysis, knowledge elicitation, decision support tools

## Abstract

Approximately 10% of newborn infants require resuscitative intervention at birth. Ideally, this care is provided by a team of expert healthcare professionals who possess exceptional cognitive, psychomotor, and communication skills. Human errors and deviations from resuscitation protocol are common and may be attributable to excessive cognitive demand experienced by the resuscitation team. Cognitive Task Analysis (CTA) is a group of methods used to assess knowledge, judgments, goals, and decision-making of expert healthcare professionals. These methods may be used during neonatal resuscitation to gain an improved understanding of the approaches used by healthcare professionals. CTA methods have been applied in many medical disciplines including neonatology. CTA has been used to identify information previously confined to the intuition of experts. This information has been used to assess, develop, and improve medical technology, clinical decision support tools (DSTs), communication structure, and training methods. Knowledge attained through CTA might be applied similarly to neonatal resuscitation, which may in turn decrease human errors, and improve patient safety.

## Introduction

During neonatal resuscitation, decisions must be made quickly and healthcare professionals (HCPs) must possess exceptional cognitive, psychomotor, and communication skills to identify problems, analyze complex scenarios, generate solutions, and refine a large amount of data into useful information under time pressure (1). This situation often leads to human errors and deviations from resuscitation protocols (2). In addition, two-thirds of preventable infant mortality/sentinel events during neonatal resuscitation are caused by poor communication or breakdown of teamwork (3–5). Therefore, understanding the cognitive, psychomotor, and communication processes used by expert HCPs might identify factors associated with human error.

Cognitive Task Analysis (CTA) can be used to examine cognitive, psychomotor, and communication processes, and to provide solutions to improve HCPs communication and education. CTA is a mechanism for capturing expertise; by breaking down complex cognitive processes that drive a set of behaviors, one can formulate comprehensive algorithmic descriptions of tasks and define task rules (6, 7). CTA is a diverse group of methods; more than 100 different systematic and scientific CTA have been described (8). For example, The Think Aloud Method is a simple and relatively well-known method of CTA. During a Think Aloud a subject matter expert (SME) verbalizes their thought process while completing a task (9). The Critical Decision Method (CDM) is another frequently used method of CTA; this method involves a retrospective interview in which participants are asked to recall a non-routine incident and are probed about decisions and judgments during that incident (10). While specific elements of each CTA approach differ, five steps are common: (1) collection of preliminary knowledge, (2) identification of knowledge representations, (3) application of focused knowledge elicitation methods, (4) analysis and verification of data acquired, and (5) formulation of results for intended applications (7). This review aims to provide an overview of previous applications of CTA in neonatology and related medical disciplines and to identify how CTA could be applied to neonatal resuscitation.

## Medical Equipment

During neonatal resuscitation, equipment must be reliable, user-friendly, and allow for easy detection of equipment flaws. Malfunctioning of equipment or user-interface issues could result in increased stress and cognitive load in HCPs, which might result in detrimental effects on patient outcomes. CTA could be used to assess how HCPs interact with medical technology, and how they recognize and cope with equipment failure.

CTA has been used to assess vital sign monitors, and incubators (11, 12). Li et al. assessed how neonatal HCPs retrieve data on vital sign monitors and identified several challenges including overwhelming alarm noises and difficulties assessing unusual events (11). This led to the development of automated aids for cardio-respiratory trend retrieval and alarm limits adjustment (11). Similarly, Ferris et al. developed blueprints of incubator systems based on CTA assessment that would better fit the needs of infants, parents, and HCPs (12). CTA identified several flaws including confusing alarm information, ambiguous commands with scale functions, unintuitive icon displays, and lack of variability in alarms with existing incubator systems, and provided solutions (12). These examples demonstrate that CTA could be used to improve neonatal resuscitation equipment for HCP use.

## Clinical Decision Support Tools

Decision support tools (DSTs) are designed to decrease the cognitive load of HCPs, improve quality of care, and decrease human errors by linking health observations with health knowledge. Though many DSTs are electronic, any tools, designs or systems which links health observations to health knowledge maybe considered a DST. DSTs cover a portion of the cognitive load previously allocated to a HCP, freeing up cognitive resources for other tasks (13). This is important as excess cognitive load has been associated with human error (14). DSTs have been developed for use in neonatal resuscitation including visual and auditory reminders to prompt interventions. Fuerch et al. assessed the impact of a visual and auditory DST and reported significantly improved mask ventilation performance (94–95% vs. 55–80% in intervention vs. control group, *p* < 0.0001) and chest compressions (82–93% vs. 71–81% in intervention vs. control group, *p* < 0.0001) during simulated neonatal resuscitations (15).

CTA has been used in the design, optimization, and integration of various clinical DSTs (16–18). Schnittker et al. used CTA to aid in the design of a DST for use during challenging airway management in adult anesthesia (16). CDM interviews and focus groups with HCPs identified that the location of airway equipment was the main contributor to successful airway management (16, 17). Based on this assessment, Schnittker et al. customized an airway equipment trolley with a strategic layout and set-up to support decision making in anesthesia (17). This approach was decided on as anesthesia team members are thought to act through a recognition-primed process that links cues and actions, the equipment was positioned as to make these cues more salient to aid in recognition and decision making (16, 17).

Despite the benefits of DSTs, the acceptance rate is often low (19). There are several barriers preventing routine use of DSTs including perceived clinical irrelevance and discordance between cognitive processes and user interface (19). CTA may be used to overcome these barriers. One example is the development of Fuzzy LOgic REspiratory Neonatal Care Expert (FLORENCE). FLORENCE is a DST for managing ventilator settings in infants with respiratory distress syndrome (18). To ensure successful acceptance and integration of FLORENCE into clinical practice, three CTA based knowledge elicitation methods—Content Analysis, CDM, and observation—were used (18). Similarly, CTA methods including Cognitive Walkthrough and Think Aloud were used to assess the interaction of neonatal HCPs with an antibiotic-prescribing support tool (20). Cognitive Walkthrough aims to simulate a user's thought process as they interact with an interface (21). These methods identified several human computer interface problems, including lack of screen cues and ambiguous icons, which were associated with excessive cognitive efforts (20). These CTA approaches could also be used to optimize the design of DST for neonatal resuscitation.

## Teamwork and Communication

During resuscitation, HCPs are reliant on team communication and a shared mental model, which serves as an exchange of patient information, education, coordination, and quality assurance. Ineffective communication can result in wasted resources, frustration, and errors, putting patients at risk (22). An improved understanding of cognitive processes involved in team communication during a neonatal resuscitation might guide the development of training and information systems to optimize team communication and performance. CTA can be used to better understand the dynamics of team communication. The benefits of CTA in analyzing team performance include understanding how teams interpret situations, make joint decisions, monitor team communication, and overcome confusion (6).

McHugh et al. used CDM in combination with direct observation to characterize communication among a multi-disciplinary critical care team (23). McHugh et al. interviewed team members individually and identified barriers (e.g., fragmentary teams, role ambiguity, external collaborators, and novice difficulty in transitioning to from tactical to strategic) and facilitators (e.g., collaborative rounds, daily goal forms, and collaborative construction) to the development of a shared understanding and multi-disciplinary collaboration (23). Similarly, CTA-based interviews with HCPs in a pediatric intensive care unit identified the teams' cognitive framework in complex pediatric patient care (24). The interviews suggested that the care teams' efforts to create a shared mental model for their patients was central to the long-term care plan of patients across shift changes and hand-overs (24). Schraagen et al. used observation-based CTA to assess team performance in a pediatric cardiac surgery care team and compared them to surgical outcomes (25). A total of 255 h of operations were observed with a 76% inter-rater agreement and a 91% inter-rater reliability of the main four teamwork categories (25). In addition, CTA has been successfully used to coach effective teamwork through simulation design (26). These studies suggest that CTA might be an alternative approach to assess team performance and knowledge retention during neonatal resuscitations as well as an effective mechanism to optimize team behaviors.

## Training

When medical experts describe medical protocols or procedures to novice learners, they typically fail to describe ~70% of the analytical and critical decisions required to successfully complete the task (27). These omissions are thought to be the experts' experiential knowledge, which is predominantly unconscious (27). CTA forces experts to consider all knowledge they use to complete a task, and in turn, make this information available to learners (28). In an assessment of education provided by surgical experts, CTA-prompted training resulted in an average of 22% more steps being described over unprompted teaching (28). By breaking down complex automated skills into bite-sized pieces, CTA could promote knowledge transfer from experts to non-experts.

### Benefits of CTA-Based Training

CTA-based instruction has been associated with an average increase of 30–45% in learning (depending on CTA methods used) when compared to traditional task analysis methods (29). A meta-analysis of CTA-based instruction reported a large effect size (Hedges's *g* = 0.871) (30). In addition, CTA-based education in surgery reported improved educational and surgical outcomes (e.g., including time, precision, and accuracy) and fewer errors compared to traditional learning methods (31).

However, the evidence of CTA-based medical education to teach neonatal trainees is limited. Crandall et al. used CDM interviews with expert Neonatal Intensive Care Unit (NICU) nurses to identify clinical symptoms for early sepsis detection (32). Overall, 36% of cues used by NICU nurses to correctly diagnose early sepsis were not reported in the medical literature or in training materials. These novel cues (e.g., “sick eyes,” poor muscle tone, and edema) were subsequently added to training material and textbooks for future nursing students (32). Similarly, CDM interviews with NICU nurses were used to identify indicators for necrotizing enterocolitis (e.g., context specific lethargy, color changes, and increased apneas) which were then shared with learners (33). CDM interviews with expert HCPs who perform neonatal resuscitation might reveal details previously overlooked or not thought of. This might be crucial information to improve learners' knowledge and improve outcomes for newborn infants.

### Expert-Novice Differences

Expert-novice differences in clinical reasoning and decision making can be identified using CTA (34). Patterson et al. used CDM interviews to understand the differences between expert and novice HCPs in their recognition of sepsis in pediatric patients and reported that experts described more hypothesis testing and violated expectations compared to novices HCPs (34). These results were then used to develop an educational tool to train novice HCPs (34).

### Simulation-Based Training

Simulations encourage experimental learning through the artificial representation of real scenarios to allow theoretical knowledge to be translated into clinical skills. This method does not put patients at risk and is independent of case availability. The benefits of simulation training in neonatal resuscitation include enhanced technical, behavioral and cognitive skill as well as improved team performance, and self-confidence (35). Regardless of learning method, skills are lost over time, which might be countered with periodic simulation training (36).

CTA could be used in simulation design to develop training scenarios and performance metrics. CTA could also help identify training needs and simulator requirements, which might result in a higher mental representation of leaners during simulation (37, 38). Cannon–Bowers et al. developed a five-step guide for simulation design using CTA. This method involves a Think Aloud followed by more focused probing to elicit critical cues, simulation deficiencies, and common errors (37). Munro et al. used a multistage CTA, called Concepts, Processes, and Principles, to elicit medical, instructional, and software expertise and integrate this into simulation design (39). During Concepts, Processes and Principles, SMEs are asked to create a gold standard list of steps involved in the completion of a task then describe critical concepts, processes, or principles that needs to be learned to explain and perform each step (29).

More recently, Pfandler et al. incorporated CTA into the design of team-based simulations. Pfandler et al. first defined all steps of the simulated procedure, then identified intra-operative technical and non-technical skills required by all involved professionals, and finally analyzed results (40). Patterson et al. used another approach involving CTA during the content and structure design of the simulations, which were then story-boarded (41). The resultant simulation was perceived as relevant and useful by learners (>70% of learners scored usefulness of simulation as >7/10) (41).

Applied CTA (ACTA) has been used to assess the impact of simulation training in pediatric extracorporeal cardiopulmonary resuscitation (ECPR) and reveal targets for further training (42). ACTA is a streamlined method of CTA used to assess aspect of expertise and present findings in an operational manner (43). This method exposed two behaviors (coordination of compression with surgical cannulation and performance of sterile compressions) which were targeted for further simulation training (42).

CTA during the design or improvement of neonatal resuscitation simulations could follow any of the methods described above. CTA could be used to determine the content and structure of scenarios, develop these training scenarios, and develop performance metrics based on the scenarios developed.

## Assessment

CTA-based assessments can be used to evaluate technical and non-technical skills in a clinical setting. Non-technical skills (i.e., communication, leadership, and situational awareness) are core competencies in clinical practice (44, 45). During neonatal resuscitations, these skills are particularly important as they have been identified as causes of human error (3–5). While no study has used CTA in the development of a non-technical assessment specific to neonatal resuscitation, these methods have been successfully used in other medical disciplines including surgery and anesthesiology (46–48). The Anesthetists' Non-Technical Skills assessment tool, for example, was developed using CTA and has been validated with high inter-rater reliability in both a clinical and simulated environment (49). Anesthetists' Non-Technical Skills assessment tool has since been used in neonatal resuscitation (50). Szulewski et al. performed a CTA of residents who completed simulation-based emergency room resuscitation exams. This approach allowed for a qualitative characterization of the cognitive processes underlying resident's crisis resource management and an examination of how these skills varied with resident's performance (51). This information could be applied to improve non-technical assessment of residents performing simulation-based resuscitation.

Similarly, CTA has been used to assess technical skills during simulations (52), scenario testing (53), and checklists (54), which are part of new surgical performance metrics. Recently, a surgical competency assessment tool to assess technical and non-technical components of transurethral resection of a bladder tumor was developed using CTA with reported feasibility, validity (*r* > 0.5, *p* < 0.01), and reliability (coefficient Phi ≥ 0.8) (55). The method used to create these tools could be adapted to assess technical and non-technical skills during neonatal resuscitation.

## Limitations of Cognitive Task Analysis

There are several limitations to CTA methods. Most studies do not describe the CTA method used in sufficient detail to reproduce the study. CTA can be a tedious and time-consuming method to obtain information (56). Furthermore, the quality of information produced is determined by the level of SME; therefore, the variability in the definition of expertise might translate to variable CTA results. Finally, due to the large number of CTA methods available, it is difficult to identify the optimal CTA method for a given clinical context.

## Application of CTA to Neonatal Resuscitation

CTA could be applied to neonatal resuscitation. Specifically, CTA could be used to improve resuscitation equipment, design clinical DSTs, assess teamwork and communication, and inform the development of training and assessment tools. As neonatal resuscitation is largely guided by standardized algorithms, CTA could also be used to study causes of algorithm deviations, analyze how algorithm adherence differs between experts and novices, and identify when experts might decide to deviate from an algorithm.

CTA could also be used to study the integration of advanced monitoring equipment into neonatal resuscitation. To supplement electrocardiogram (ECG) and pulse oximetry (SpO_2_) currently recommended by international neonatal resuscitation guidelines, additional monitoring including near cerebral infrared spectroscopy (NIRS) and respiratory function monitoring (RFM) are being investigated (57, 58). CTA could be used to examine how HCPs integrate information provided by these monitors into established clinical decision pathways. This knowledge could be used to design monitor displays to present information in a manner that best fits the pre-existing cognitive framework of HCPs, thus optimizing usability while minimizing additional cognitive demands. CTA could also be used to guide the design of a DST that integrates ECG, SpO_2_, NIRS, RFM with standard neonatal resuscitation algorithms.

Neonatal resuscitation presents unique challenges to the application of CTA methods. Given the fast-paced and demanding nature of neonatal resuscitation, it may not be feasible to use CTA methods that occur during actual resuscitations (e.g., Think Aloud), as this may increase cognitive burden and compromise clinical performance. Instead, CTA methods that must be conducted during task completion could be performed during simulated neonatal resuscitation. Alternatively, videos recordings of resuscitations could be used to prompt recall. Mobile eye-tracking glasses have been used to analyze visual attention of HCPs during neonatal resuscitation (59). These glasses use reflected infrared light to track pupillary movements while simultaneously recording video from the wearer's viewpoint. The resulting video indicates the wearer's visual focus during the performance of clinical tasks (e.g., what they were looking at when performing bag-mask ventilation). Thus, eye-tracking recordings can be used to provide an information-rich prompt for the recall of knowledge, cognitive tasks, and decision-making after an actual neonatal resuscitation event. Recently, Katz et al. used the Think Aloud method along with mobile eye-tracking glasses to analyze how neonatal HCPs interact with respiratory function monitors during simulated positive pressure ventilation. This technique allowed the authors to confirm that HCPs cognitive attention corresponded with their visual attention (60).

Given the diverse physical and social environments in which neonatal resuscitation occur and the variety of HCP roles required to complete this task, it is unlikely that the cognitive pathways exposed using CTA in one context will be generalizable to all settings. Knowledge elicited from an HCP practicing in a tertiary care center may not be generalizable to an HCP practicing in a low-resource setting. Similarly, SME from different disciplines (physician, nursing, respiratory therapy, midwifery, etc.) may offer significantly different perspectives despite applying the same CTA methods in the same clinical context. CTA that examines neonatal resuscitation across settings and roles is therefore needed to accurately inform improvements to neonatal resuscitation equipment, DST, teamwork, and training.

## Conclusions

CTA is a method to elicit expertise, which then can be applied to improve medical technology, clinical DSTs, teamwork, and training. The application of CTA to neonatal resuscitation has the potential to improve knowledge in novice learners, reduce human errors, and improve outcomes for newborn infants.

## Author Contributions

GS, BL, and EZ: conception and design, collection and assembly of data, analysis and interpretation of the data, drafting of the article, critical revision of the article for important intellectual content, and final approval of the article.

### Conflict of Interest Statement

The authors declare that the research was conducted in the absence of any commercial or financial relationships that could be construed as a potential conflict of interest.

## Abbreviations

ACTA: Applied Cognitive Task Analysis
CDM: Critical decision method
DST: Decision Support Tool
ECPR: Pediatric Extracorporeal Cardiopulmonary Resuscitation
FLORENCE: Fuzzy LOgic REspiratory Neonatal Care Expert
HCP: Health care professionals
NICU: Neonatal Intensive Care Unit
SME: Subject matter experts.
